# Renal Functional Reserve–Informed Personalized Renoprotection in Chronic Kidney Disease: A Proposed Extension of the KDIGO CGA Framework

**DOI:** 10.3390/biomedicines14071478

**Published:** 2026-06-29

**Authors:** Dmytro D. Ivanov, Anatoliy I. Gozhenko, Volodymyr V. Bezruk, Mariia D. Ivanova

**Affiliations:** 1 Department of Nephrology and Extracorporeal Therapies, Bogomolets National Medical University, 01601 Kyiv, Ukraine; 2 State Enterprise “Ukrainian Research Institute of Transport Medicine”, Kanatna Street, 92, 65039 Odesa, Ukraine; prof.gozhenko@gmail.com; 3 Department of Pediatrics, Neonatology and Perinatal Medicine, Bukovinian State Medical University, 58002 Chernivtsi, Ukraine; vvladimyrbezruk@gmail.com; 4 Department of Pathology, University Vita-Salute San Raffaele, 20132 Milan, Italy; mesangium88@gmail.com

**Keywords:** chronic kidney disease, renal functional reserve, hyperfiltration, albuminuria, eGFR, SGLT2 inhibitors, RAAS blockade, finerenone, mineralocorticoid receptor antagonists, precision nephrology, tubuloglomerular feedback

## Abstract

The Kidney Disease: Improving Global Outcomes (KDIGO) CGA framework remains the essential basis for chronic kidney disease (CKD) classification, risk stratification, and guideline-based therapy. However, eGFR and albuminuria do not always explain the physiological mechanism maintaining the current filtration level or the heterogeneity of treatment responses. This narrative review proposes a hypothesis-generating functional–hemodynamic extension of KDIGO CGA that incorporates renal functional reserve (RFR), blood pressure, volume status, proteinuria phenotype, and selected tubular markers. RFR is discussed as a dynamic stress test of nephron reserve rather than as a replacement for eGFR or albuminuria. A low, zero, or negative RFR may suggest reserve exhaustion or relative hyperfiltration, but its interpretation depends on standardized testing conditions and clinical context. We distinguish established evidence-based therapy—RAAS blockade in albuminuric or hypertensive CKD, SGLT2 inhibition for kidney and cardiorenal protection, and non-steroidal MRA therapy in selected patients—from conceptual sequencing hypotheses such as RAASi-prioritized, SGLT2i-prioritized, early dual, or staged triple renoprotection. The review also summarizes albuminuria as a two-compartment phenomenon involving both glomerular passage and proximal tubular handling of filtered proteins. The proposed framework is not a validated treatment algorithm. It is intended to support physiological phenotyping, interpretation of early eGFR changes, and the design of prospective studies that test whether RFR adds independent prognostic or therapeutic value beyond KDIGO CGA.

## 1. Conceptual Framework: From KDIGO CGA to Functional Renoprotection

The classical KDIGO model describes CKD along three axes: cause, GFR, and albuminuria. In daily practice, this model identifies risk category, surveillance intensity, the need for additional evaluation, and broad therapeutic direction [1,2]. However, when clinicians select renoprotective treatment, the practical question is often more granular: which drug should be introduced first, how rapidly should therapies be combined, when should treatment be intensified, and in what sequence should RAAS inhibitors, SGLT2 inhibitors, and non-steroidal mineralocorticoid receptor antagonists (ns-MRAs) be used?

**Figure 1:**
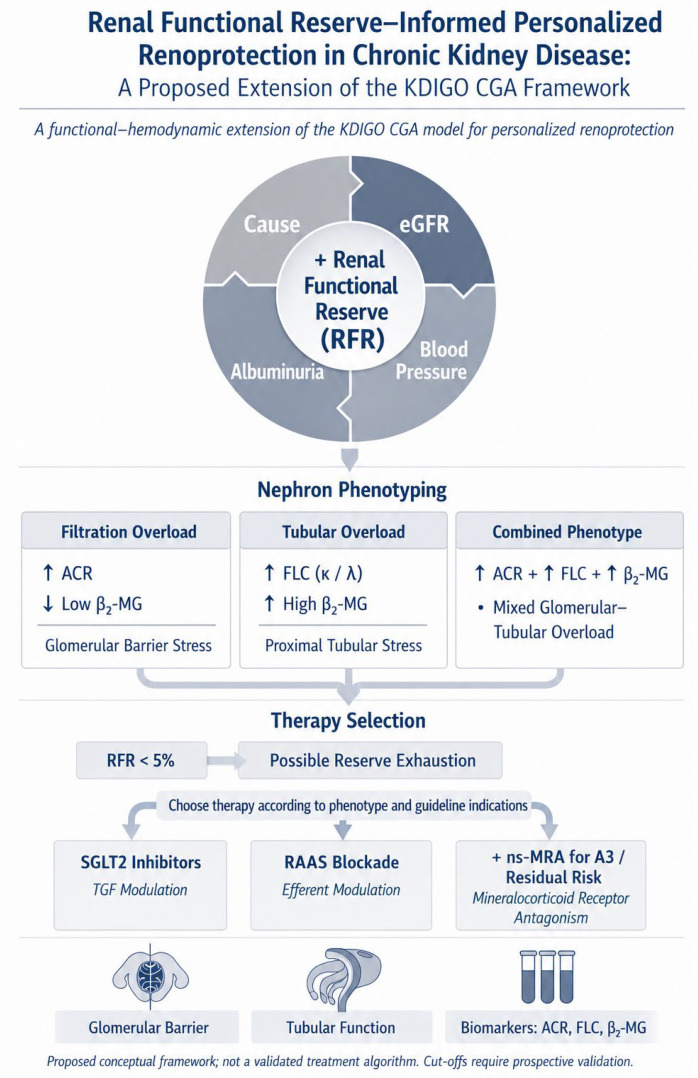
Proposed conceptual framework for renal functional reserve-informed extension of the KDIGO CGA framework. The figure is intended for hypothesis generation and should not be interpreted as a validated treatment algorithm.

The overall proposed framework is summarized in Figure 1.

We propose that KDIGO CGA should be regarded not as a final drug-selection algorithm, but as the necessary first layer. A second layer should include renal functional reserve, blood pressure, volume status, eGFR slope, the phenotype of proteinuria or albuminuria, and additional tubular markers. This extension builds on the previously proposed idea of adding blood pressure to CKD prognosis based on GFR and albuminuria categories [3] and translates it into a more functional model by placing RFR as a dynamic stress test of nephron reserve [4,5,6]. The model can therefore be expressed as:

Cause + GFR + Albuminuria + Renal Functional Reserve + Blood Pressure.

This sequence reflects the logic of the present article. Structural and functional risk is first established using KDIGO; the nephron reserve is then assessed functionally; and blood pressure is subsequently used as a critical hemodynamic modifier that determines drug choice, dose, titration speed, and the safety of combination therapy. We therefore do not propose a new antihypertensive algorithm. Rather, we suggest using blood pressure as an additional clinical coordinate that modifies the sequencing of kidney-protective therapy.

This additional functional-hemodynamic layer may identify the dominant pathophysiological node: efferent-glomerular, afferent/TGF-mediated, proximal tubular, postglomerular-peritubular, inflammatory-fibrotic, or mixed. Therapy can then be individualized: RAASi-prioritized for a pressure-driven or albuminuric phenotype; SGLT2i-prioritized for a TGF-mediated hyperfiltration phenotype with normal or low-normal blood pressure; early dual therapy for mixed phenotypes; and staged triple therapy for high residual risk when functional reserve is preserved [7,8,9,10].

The purpose of this concept is not to revise KDIGO, but to build a physiological extension above it. Such an extension may be particularly useful when eGFR and urinary albumin-to-creatinine ratio (UACR) provide an incomplete or misleading picture: normal eGFR despite reduced nephron mass, low albuminuria despite marked tubular overload, elevated blood pressure with minimal albuminuria, a pronounced eGFR dip after therapy initiation, or uncertainty about which disease-modifying drug should be introduced first.

### Added Value Beyond KDIGO CGA: Independent, Non-Redundant, and Hypothesis-Generating Information

The proposed extension is intended to add information that is not fully captured by the CGA heat map. KDIGO CGA defines the risk category and indicates broad evidence-based therapeutic directions, but it does not directly test whether the remaining nephron mass has recruitable functional reserve, whether total eGFR is being maintained by increased single-nephron workload, or which hemodynamic compartment is most vulnerable to pharmacological unloading. RFR, therefore, should not be interpreted as a replacement for eGFR or albuminuria, but as a dynamic functional stress test that may reveal a latent state of reserve exhaustion.

Blood pressure is likewise not a duplicate of albuminuria or eGFR. It captures the systemic and intrarenal pressure environment within which nephroprotective drugs act. Two patients with identical eGFR and UACR may differ markedly in nocturnal blood pressure profile, sodium sensitivity, volume status, pulse pressure, and vulnerability to an early eGFR dip. In this sense, RFR and blood pressure may provide non-redundant therapeutic information: RFR indicates whether functional reserve is present, whereas blood pressure and volume status help determine which intervention can be introduced safely and in what sequence.

At present, the incremental prognostic value of RFR beyond KDIGO CGA has not been established in large prospective cohorts. For this reason, the model is deliberately framed as a conceptual and hypothesis-generating extension. Its clinical value should be tested by determining whether RFR and blood pressure improve the prediction of eGFR slope, albuminuria response, treatment tolerability, magnitude of acute eGFR dip, and long-term kidney outcomes after adjustment for established CGA categories.

## 2. Blood Pressure in the Proposed Model: A Risk Criterion, Not a Separate Treatment Algorithm

Blood pressure is not formally a CKD stage within the KDIGO CGA system. Clinically, however, it is a third major vector of CKD progression alongside eGFR and albuminuria. Systemic pressure, intraglomerular pressure, pulse pressure, sodium status, volume load, nocturnal non-dipping, and vascular stiffness may profoundly modify both progression risk and the tolerability of renoprotective therapy [3,11].

In this review, blood pressure is used not as a stand-alone treatment target but as a hemodynamic modifier of the proposed algorithm. Comprehensive blood pressure management requires a separate discussion, including antihypertensive combinations, standardized office measurement, home monitoring, ambulatory blood pressure monitoring, nocturnal profiles, and arterial stiffness. For this reason, we do not place blood pressure above RFR in the conceptual hierarchy, but rather consider it after RFR as the parameter that determines therapeutic sequencing and safety.

The practical reference point remains the KDIGO 2021 recommendation that adults with CKD and elevated blood pressure should be treated to a target systolic blood pressure below 120 mmHg, when tolerated and using standardized office measurement [11]. This does not imply that every patient with CKD should undergo aggressive pressure reduction at any cost. In patients with low RFR, advanced age, diuretic therapy, heart failure, or hypovolemia risk, excessive blood pressure reduction may amplify an eGFR dip and limit the feasibility of combined renoprotection [12].

In the proposed algorithm, RFR answers the question: Is the kidney operating with reserve, or already at its functional limit? Blood pressure answers a different question: which route for lowering intrarenal stress is safer, and what should be introduced first—efferent/postglomerular modulation with RAAS blockade, afferent/TGF modulation with SGLT2 inhibition, or cautious staged combination therapy?

### Interpreting Blood Pressure Targets Across RFR States

The systolic blood pressure target below 120 mmHg cited in this review should be understood as a reference derived from KDIGO 2021 for adults with CKD and elevated blood pressure when standardized office measurement is used, and treatment is tolerated [11]. It should not be applied mechanically to every phenotype discussed here. The patient groups considered in this review—solitary kidney, ADPKD, transplant recipients, advanced age, low or negative RFR, intensive diuretic therapy, heart failure, or high hypovolemia risk—may differ substantially from the populations in which intensive blood-pressure targets were tested.

Within the proposed model, blood pressure has two roles. First, it remains a guideline-based treatment target when hypertension is present. Second, it acts as a hemodynamic safety modifier when renoprotective therapy is introduced. In a patient with preserved RFR and stable volume status, blood pressure reduction and combination therapy may be titrated more confidently. In a patient with zero or negative RFR, low-normal blood pressure, or high volume sensitivity, the same target should be pursued cautiously, with slower titration and closer monitoring of symptoms, urea, creatinine, potassium, body weight, and albuminuria.

Thus, blood pressure does not override KDIGO indications or independently mandate a specific drug sequence. Rather, it contextualizes whether RAASi-prioritized, SGLT2i-prioritized, or staged combination therapy can be introduced safely in a given hemodynamic state.

## 3. Contemporary Therapeutic Context: Three Core Classes of Renoprotection

Current evidence has moved nephrology toward a model of multiple foundational renoprotective therapies. The first component is RAAS blockade, which remains central in proteinuric and albuminuric CKD, particularly in diabetes and hypertension. The second component is SGLT2 inhibition, which reduces the risk of CKD progression across a wide range of eGFR and albuminuria, including in patients without diabetes. The third component is ns-MRA therapy, especially finerenone, which reduces inflammatory-fibrotic and cardiovascular residual risk in patients with type 2 diabetes, CKD, and persistent albuminuria.

The CREDENCE, DAPA-CKD, and EMPA-KIDNEY trials established that SGLT2 inhibitors reduce CKD progression and adverse cardiorenal outcomes [13,14,15]. Meta-analyses indicate that SGLT2i reduce the risk of CKD progression by approximately 30–40% in patients with eGFR below 60 mL/min/1.73 m^2^ and/or severe albuminuria, with benefit observed across different baseline eGFR and UACR strata [16,17].

Most major SGLT2i trials were conducted on a background of established or recommended RAAS blockade when tolerated [18], and real-world audits and network meta-analyses continue to evaluate how RAASi, SGLT2i, and aldosterone/mineralocorticoid-pathway therapies are combined in practice [19,20]. Therefore, in conventional evidence-based pathways, SGLT2i are frequently added to stable RAASi therapy. Yet real-world practice is broader than randomized trial design. A patient may have low blood pressure, A1 albuminuria, absent functional reserve, high hypovolemia risk, solitary kidney, obesity, diabetes without albuminuria, or early CKD with preserved eGFR. In these scenarios, the question of where to start remains clinically relevant.

RAASi, SGLT2i, and ns-MRAs should not be viewed as competing therapies. They address different components of CKD pathophysiology. RAASi primarily reduces efferent arteriolar tone, intraglomerular pressure, and may improve postglomerular peritubular perfusion [10,21]. SGLT2i directly reduces proximal tubular sodium and glucose reabsorption, increases sodium delivery to the macula densa, restores tubuloglomerular feedback, increases afferent arteriolar tone, and reduces intraglomerular pressure [9,22,23]. ns-MRAs reduce mineralocorticoid-mediated inflammation, fibrosis, and vascular-cardiorenal residual risk [24,25,26]. Personalized treatment should therefore depend on which mechanism is dominant in the individual patient.

## 4. Albuminuria as a Two-Compartment Phenomenon

Albuminuria is best interpreted as a two-compartment signal rather than as a direct one-to-one measurement of glomerular leak. The first compartment is glomerular: albumin passage depends on intraglomerular pressure, podocyte integrity, endothelial glycocalyx, the glomerular basement membrane, and size/charge selectivity. The second compartment is tubular: filtered albumin and low-molecular-weight proteins are retrieved and metabolically processed by the proximal tubule through megalin–cubilin-dependent endocytosis and lysosomal pathways [27,28,29,30,31,32,33,34,35,36,37,38].

The relative contribution of glomerular filtration and tubular reabsorption remains debated, particularly regarding the magnitude of physiological albumin filtration in humans. Observations in rare inherited tubular disorders, such as congenital megalin deficiency, are useful as human proof-of-principle models showing that tubular handling can substantially shape urinary protein composition; however, these disorders should not be overgeneralized to the entire CKD population [29,38].

For the present framework, the exact amount of albumin filtered under physiological conditions is less important than the clinical principle: the same UACR value may arise from different combinations of glomerular barrier stress, intraglomerular pressure, peritubular perfusion, proximal tubular workload, and endocytic capacity. Therefore, this section should be read as a mechanistic and hypothesis-generating interpretation, not as a claim that tubular mechanisms dominate in all forms of CKD.

For renoprotective sequencing, the key implication is that an antialbuminuric response may reflect reduced glomerular pressure, improved postglomerular/peritubular perfusion, reduced proximal tubular workload, or a combination of these mechanisms. RAASi and SGLT2i should therefore be viewed as potentially complementary rather than interchangeable: RAASi mainly target the efferent/postglomerular compartment, whereas SGLT2i mainly reduce proximal sodium-glucose transport and restore tubuloglomerular feedback.

## 5. KDIGO Albuminuria and Terminological Clarification

Using the KDIGO classification, urinary albumin-to-creatinine ratio should be interpreted as follows: UACR below 3 mg/mmol corresponds to A1, normal or mildly increased albuminuria; UACR 3–30 mg/mmol corresponds to A2, moderately increased albuminuria; and UACR above 30 mg/mmol corresponds to A3, severely increased albuminuria, where clinically significant glomerular or podocyte injury and/or marked intraglomerular hypertension are more likely.

Albuminuria and non-selective proteinuria should not be conflated, and the albumin-to-total-protein ratio may help distinguish glomerular from non-glomerular sources in selected clinical settings [39,40]. Even when UACR exceeds 30 mg/mmol, the measurement still quantifies albumin rather than the full spectrum of urinary proteins. Assessment of non-selectivity requires additional tests: total urine protein, protein-to-creatinine ratio, the albumin-to-total-protein ratio, IgG, transferrin, alpha1-microglobulin, beta2-microglobulin, retinol-binding protein, and, when appropriate, urine protein electrophoresis.

Nevertheless, A3 albuminuria is a strong clinical signal. It may reflect a glomerular barrier lesion, podocyte injury, endothelial or glycocalyx dysfunction, or pronounced intraglomerular hypertension. In such cases, RAASi generally remains the most physiologically grounded first-line therapy, with early SGLT2i addition and, when indicated, ns-MRA therapy.

### Practical Phenotyping of Proteinuria Before Renoprotective Drug Selection

Before selecting a renoprotective sequence, it is important to establish whether persistent proteinuria is glomerular, tubular, mixed, postglomerular, or physiological/orthostatic. This step is frequently overlooked when only UACR or a dipstick is available. Bökenkamp emphasized several practical principles: dipsticks detect mainly albumin; protein concentration varies with urine flow; a first-morning urine sample is essential when orthostatic proteinuria is suspected; and 24 h collections should be interpreted cautiously because incomplete or prolonged collection is common [41].

Orthostatic proteinuria is characterized by absent proteinuria in the recumbent position, typically in the first-morning void, with proteinuria appearing later during the day. It is common in adolescents and young adults, has an excellent prognosis, and should not be misclassified as progressive glomerular CKD [41,42]. Conversely, persistent first-morning proteinuria, abnormal sediment, hypoalbuminemia, edema, reduced eGFR, or hypertension should prompt further evaluation.

The albumin-to-total-protein ratio provides an additional practical clue. A predominantly albuminuric pattern suggests a glomerular component, whereas a low albumin fraction with marked low-molecular-weight proteinuria suggests tubular proteinuria. Intermediate values may reflect mixed or postglomerular sources [39]. This distinction is directly relevant to the proposed algorithm because the dominant site of protein handling determines whether the therapeutic target should be glomerular pressure, proximal tubular workload, peritubular perfusion, or residual inflammatory-fibrotic risk [39,40].

The practical phenotyping approach is summarized in Table 1.

## 6. Tubular Proteinuria, Megalin–Cubilin, and Low-Molecular-Weight Markers

The proximal tubule is equipped with a high-capacity endocytic system. Megalin, cubilin, ClC-5, the early and late endosomal compartments, and the lysosomal pathway work together to reclaim low-molecular-weight proteins and a limited amount of filtered albumin [31,32,33,43,44,45,46,47]. When this system is genetically or functionally impaired, urinary protein composition changes even when the glomerular barrier is not the primary lesion.

Dent disease provides a useful model. ClC-5 or OCRL-related proximal tubular dysfunction causes low-molecular-weight proteinuria, hypercalciuria, nephrocalcinosis, nephrolithiasis, and progressive CKD in a subset of patients [44,46,48]. In such disorders, the magnitude of total proteinuria may be misleading if interpreted as a purely glomerular process. The urine is enriched with low-molecular-weight proteins, whereas albuminuria is often modest relative to total protein.

This is relevant to personalized renoprotection because tubular overload may coexist with glomerular stress; the composition of urinary proteins can therefore be more informative than total protein alone [34,35,36,37]. A patient may have A1 or A2 albuminuria but still have marked proximal tubular stress, reflected by urinary beta2-microglobulin, alpha1-microglobulin, retinol-binding protein, cystatin C, or free light chains. In such a phenotype, eGFR and UACR alone may underestimate the degree of nephron stress.

## 7. Free Light Chains and Beta2-Microglobulin as Indicators of Nephron Overload

Free light chains (FLCs) may be viewed as sensitive indicators of which nephron compartment is overloaded, particularly when interpreted together with eGFR, tubular markers, and functional testing [49,50]. In early CKD, especially when eGFR is still normal or only mildly reduced, increased urinary FLCs may reflect proximal tubular overload, hypoxia, and impaired megalin–cubilin-mediated reabsorption. In more advanced CKD, reduced filtration and systemic accumulation may contribute more substantially. The pattern of FLC increase can therefore provide indirect information about whether the limiting node is primarily tubular, glomerular, or mixed.

Beta2-microglobulin (beta2-MG; approximately 11.8 kDa) is freely filtered and almost completely reabsorbed by the proximal tubule. Increased urinary beta2-MG is a sensitive marker of proximal tubular dysfunction and may reflect structural or functional tubular injury independent of GFR [51]. In the context of RFR testing, elevated beta2-MG may suggest that absent reserve is not merely a glomerular pressure problem but also a sign of proximal tubular overload or tubulointerstitial stress.

Combining UACR, FLCs, beta2-MG, and RFR may help determine whether the nephron is limited by the glomerular filtration barrier, the proximal tubular reabsorptive system, or both. This can guide whether RAASi, SGLT2i, or combination therapy is most physiologically appropriate.

Practical limitations must be acknowledged. Tubular markers are not universally available, assays may be costly, pre-analytical handling can influence results, and reference intervals are not harmonized across laboratories. Urinary beta2-MG is unstable in acidic urine, and FLC interpretation may be affected by systemic production as well as kidney handling. For this reason, tubular markers should be used as supportive phenotyping tools rather than mandatory routine tests until broader standardization and outcome validation are available.

The choice of FLCs and beta2-MG in this framework is intentional rather than exhaustive. They are emphasized because they are closely linked to filtration load and proximal tubular reabsorptive capacity, and because they are more directly connected to the two-compartment protein-handling concept discussed above. Other CKD biomarkers, including NGAL, KIM-1, urinary calprotectin, MCP-1, EGF, TNF receptors, uromodulin, extracellular vesicle markers, metabolomic signatures, and multi-marker panels, have been investigated for CKD progression, tubular injury, inflammation, fibrosis, and tissue repair [52,53,54].

These broader biomarkers may ultimately enrich RFR-informed phenotyping, but they are not yet integrated into routine nephrology decision-making and were therefore not used as core elements of the proposed algorithm. Their added value should be assessed in prospective studies together with RFR, UACR, eGFR slope, and blood pressure.

The conceptual interpretation of nephron overload markers is summarized in Table 2.

## 8. Limitations of eGFR: Why Normal eGFR Does Not Exclude CKD or Hyperfiltration

KDIGO uses eGFR and albuminuria as core axes of CKD prognosis, and both lower eGFR and higher albuminuria predict adverse kidney outcomes across populations [55]. Yet eGFR represents the sum of filtration by all functioning nephrons, not the workload imposed on each individual nephron. A patient with reduced nephron number may maintain a normal total eGFR through increased single-nephron GFR. In such circumstances, normal eGFR may conceal relative hyperfiltration and a diminished capacity to adapt to additional stress [8,56,57,58,59].

Absolute hyperfiltration is usually defined as GFR above the expected range for age, sex, and body size. Relative hyperfiltration is more difficult to detect. It may occur when total eGFR is normal or mildly reduced, while the remaining nephrons already operate at high single-nephron filtration. This situation is relevant in diabetes, obesity, insulin resistance, solitary kidney, nephron loss after nephrectomy or congenital reduction in nephron mass, ADPKD, transplant recipients, and some glomerular diseases [59,60,61,62,63].

Creatinine-based equations, including CKD-EPI and FAS, are valuable but cannot identify single-nephron hyperfiltration. The FAS equation adjusts creatinine to age- and sex-specific Q values and can provide age-adapted assessment, while CKD-EPI remains a widely used creatinine-based estimator; neither directly measures nephron workload or reserve [56,57,64]. Cystatin C or combined creatinine-cystatin C equations may improve the accuracy of GFR estimation [58], yet they still do not replace functional testing of renal reserve.

## 9. Illustrative Calculation: Apparent Stability of eGFR and the Need for Functional Assessment

Consider a teaching example of a 61-year-old man with serum creatinine 1.36 mg/dL and CKD-EPI eGFR approximately 56 mL/min/1.73 m^2^. A simplified FAS calculation without age correction may misleadingly suggest a higher value. However, the correct FAS equation in adults older than 40 years incorporates the age multiplier 0.988^(age − 40). With Scr/Q approximately 1.36/0.90 = 1.51, the first part of the equation yields approximately 71 mL/min/1.73 m^2^; applying the age multiplier 0.988^21 gives approximately 55 mL/min/1.73 m^2^. Thus, CKD-EPI and FAS converge, supporting CKD G3a if persistence is documented.

This example does not demonstrate absolute hyperfiltration. Rather, it illustrates a more important point: neither CKD-EPI nor FAS answers whether the remaining nephron mass has functional reserve. If protein or amino acid loading increases GFR, reserve is preserved; if GFR fails to increase or falls, the kidney may already be functioning near its adaptive limit. RFR therefore provides information that eGFR equations cannot supply.

## 10. Renal Functional Reserve as a Stress Test of Nephron Reserve

### 10.1. Operational Definition and Practical Standardization of RFR Testing

For the purposes of this review, RFR is operationally defined as the absolute or relative increase in GFR after a standardized renal stressor compared with a basal pre-stimulus value. When measured GFR is available, iohexol, iothalamate, inulin, DTPA, or comparable clearance methods are preferable. In routine practice and in hypothesis-generating protocols, creatinine-based or cystatin C-based estimates may be used as pragmatic surrogates, but their limitations must be explicitly acknowledged, particularly when short-term changes are small.

The stressor should be prespecified. Published protocols have used oral protein loading, cooked meat meals, amino acid infusion, or dopamine-based approaches; protein or amino acid loading is generally the most physiologically intuitive for clinical nephrology. A practical research protocol should define fasting or standardized dietary conditions, baseline sampling, protein or amino acid type and dose, hydration instructions, posture/activity, timing of post-load measurements, and whether plasma clearance, urine clearance, creatinine, cystatin C, or measured GFR is used. Many protocols assess the response over approximately 2–4 h after the load, but timing should remain protocol-specific and reproducible within a study. Results should be reported both as an absolute change in mL/min/1.73 m^2^ and as a percentage change from baseline.

Reproducibility requires control of pre-analytical conditions. Patients should ideally be clinically stable, free of acute illness, and without recent major changes in diuretic dose, RAAS blockade, SGLT2 inhibition, non-steroidal MRA therapy, non-steroidal anti-inflammatory drugs, dietary protein intake, or sodium intake. A standardized sodium intake or at least documentation of usual sodium exposure is important because sodium balance modifies tubuloglomerular feedback, RAAS tone, extracellular volume, and renal vascular reactivity. The same GFR estimation or measurement method should be used before and after the stressor, and when possible, repeated testing should be performed in the same laboratory and under similar conditions.

Several biological variables may reduce RFR or alter its interpretation. Aging reduces nephron number and renal vascular reserve; diabetes may increase proximal tubular sodium-glucose reabsorption and alter TGF; proteinuria may reflect glomerular barrier stress and secondary tubular overload; obesity and high-protein diet may increase basal filtration; and concurrent pharmacotherapy may shift afferent, efferent, and tubular sodium-handling responses. Consequently, RFR should not be considered a universal numeric threshold in isolation. In the present framework, low, zero, or negative RFR identifies a need for functional phenotyping, not an automatic prescription decision.

A pragmatic RFR testing protocol for research or specialized clinical phenotyping should include: (1) a clinically stable patient without intercurrent illness; (2) documented background therapy, especially diuretics, RAASi, SGLT2i, ns-MRA, NSAIDs, and recent dose changes; (3) standardized or recorded sodium and protein intake before testing; (4) baseline blood pressure, body weight, hydration status, serum creatinine, urea, potassium, and UACR; (5) a prespecified renal stressor, preferably oral protein/meat load or amino acid load; (6) repeated post-load GFR assessment over a defined interval, commonly 2–4 h depending on the protocol; and (7) reporting of both absolute and relative RFR.

A practical checklist for RFR testing and reporting is provided in Table 3.

### 10.2. Hemodynamic, Sodium, and Volume-Dependent Confounders

RFR, blood pressure, and early eGFR changes are highly sensitive to intravascular volume, sodium balance, RAAS activity, and renal perfusion pressure. Hypovolemia, aggressive diuretic therapy, low sodium intake, heart failure decompensation, febrile illness, or recent initiation of RAASi or SGLT2i may all blunt the RFR response or exaggerate an acute eGFR dip. Conversely, high sodium intake, volume expansion, obesity, hyperglycemia, and high protein intake may increase basal filtration and thereby reduce the apparent recruitable reserve.

This confounding is not a weakness of the concept but a reason for standardization. In clinical studies, RFR assessment should be accompanied by documentation of office and preferably ambulatory blood pressure, body weight, edema status, diuretic use, dietary sodium and protein exposure, glycemic state, serum urea, serum creatinine, potassium, albuminuria, and urinary tubular markers. In clinical practice, a low RFR result should be interpreted cautiously if obtained during unstable volume status or shortly after major medication changes.

The same principle applies to short-term eGFR changes after therapy initiation. A modest early eGFR dip with lower UACR and stable urea may indicate beneficial hemodynamic unloading, whereas a progressive decline accompanied by rising urea, worsening albuminuria, hypotension, or volume depletion should prompt reassessment of the presumed phenotype, drug sequence, and volume-sodium state.

Renal functional reserve is the capacity of the kidney to increase GFR in response to a physiological or pharmacological stimulus, most commonly protein or amino acid loading. It represents the difference between basal GFR and the maximally achievable filtration response under standardized conditions [4,5,65,66].

A preserved RFR suggests that functioning nephrons retain adaptive capacity. A low, zero, or negative RFR suggests that the kidney is already operating close to maximal filtration, or that it cannot appropriately recruit additional filtration because of vascular stiffness, nephron loss, tubulointerstitial injury, or hemodynamic maladaptation. In the present model, a zero or negative RFR is interpreted as a marker of an actionable hyperfiltration phenotype when the clinical context supports this interpretation.

This does not mean that RFR alone diagnoses the mechanism of injury. Instead, RFR identifies whether a reserve exists. The mechanism must then be inferred from UACR, protein phenotype, tubular markers, blood pressure, eGFR slope, diabetes, obesity, heart failure, solitary kidney, ADPKD, transplant status, and response to initial therapy. RFR is thus a functional gatekeeper rather than a stand-alone biomarker.

### 10.3. Interpretation of RFR Cut-Off Values

The cut-off values used in this manuscript are conceptual and should not be interpreted as universally validated clinical thresholds. A preserved RFR generally indicates a measurable ability to recruit additional filtration after a standardized stimulus. A low RFR suggests limited recruitability; a zero RFR indicates no measurable increase from baseline; and a negative RFR indicates a paradoxical fall in GFR after stress. The <5% value used in the proposed algorithm is a pragmatic hypothesis-generating threshold intended to flag possible reserve exhaustion, not a guideline-endorsed treatment boundary. It was selected because values close to zero are unlikely to reflect robust recruitable reserve, but the exact boundary may differ by stressor, GFR method, age, sodium state, CKD etiology, and background therapy. Therefore, the manuscript uses these categories as research stratification tools and clinical reasoning prompts, not as treatment mandates. Prospective studies must determine which RFR categories best predict eGFR slope, albuminuria response, early eGFR dip, tolerability, and long-term kidney outcomes.

## 11. What Happens to the Nephron During Hyperfiltration?

Hyperfiltration is not a benign increase in filtration. It reflects maladaptive recruitment of glomerular hemodynamics, tubular transport, oxygen consumption, and vascular tone. In reduced nephron mass, the remaining nephrons increase single-nephron GFR to preserve total filtration. Initially compensatory, this state may increase glomerular capillary pressure, podocyte stress, protein leak, proximal tubular workload, peritubular hypoxia, and inflammatory-fibrotic signaling [7,8,67,68,69,70,71].

If RFR is preserved, a further increase in GFR can still be elicited after a standardized load. If RFR is absent or negative, the nephron may already be operating at or beyond its adaptive ceiling. Such a patient may be particularly sensitive to any drug that alters afferent or efferent tone, volume status, or tubular sodium handling. This explains why the same therapeutic class may produce a desired eGFR dip in one patient and an excessive decline or albuminuria worsening in another.

## 12. Two Mechanisms of the Antialbuminuric Effect of RAAS Blockade

The classical explanation for the antialbuminuric effect of RAAS blockade is efferent arteriolar vasodilation, reduced intraglomerular pressure, and reduced albumin filtration. This mechanism is well established and underlies KDIGO recommendations for ACE inhibitors or angiotensin receptor blockers in patients with A2 or A3 albuminuria, particularly in diabetes and hypertension [1,21].

However, this may not be the only mechanism. Peritubular capillaries are downstream of the glomerulus and arise from the efferent arteriole. Excessive efferent vasoconstriction may impair postglomerular perfusion. RAAS activation may therefore sustain both intraglomerular hypertension and peritubular hypoperfusion, thereby promoting tubulointerstitial hypoxia and impairing tubular reabsorption of filtered proteins [10,67,68,71,72].

From this perspective, RAAS blockade may reduce albuminuria through two pathways: by reducing albumin passage across the glomerular barrier through lower intraglomerular pressure, and by improving downstream peritubular perfusion, reducing proximal tubular ischemia, and restoring megalin–cubilin-mediated reabsorption and degradation of filtered proteins.

This distinction matters for personalized therapy. If albuminuria is primarily driven by glomerular pressure and barrier leak, RAASi and SGLT2i partly converge on the common final pathway of lowering intraglomerular pressure. If peritubular ischemia and impaired tubular protein handling contribute substantially, RAASi and SGLT2i become particularly complementary: RAASi may improve the efferent-peritubular component, while SGLT2i reduces proximal sodium-glucose transport and restores TGF.

## 13. SGLT2i: Direct Tubular Target and Afferent Hemodynamic Output

The expression “SGLT2i-prioritized in a tubular-hyperfiltration phenotype” may be imprecise. A more accurate term is “proximal tubular/TGF-mediated hyperfiltration phenotype” or simply “TGF-mediated phenotype.” SGLT2 inhibitors act directly in the proximal tubule by inhibiting SGLT2-dependent sodium and glucose reabsorption. Their main anti-hyperfiltration hemodynamic output, however, is mediated by increased sodium delivery to the macula densa, restoration of tubuloglomerular feedback, increased afferent arteriolar tone, and reduced intraglomerular pressure [9,22,23,73,74,75].

Thus, SGLT2i should not be described as drugs that directly restore megalin–cubilin-mediated protein endocytosis. Their action is to reduce proximal sodium-glucose transport work, lower proximal tubular oxygen demand, restore TGF, and correct maladaptive hyperfiltration. RAASi, in contrast, more directly influences the efferent/postglomerular compartment and may affect peritubular perfusion.

This distinction is clinically important. If SGLT2i initiation produces an eGFR dip accompanied by lower UACR, the response may represent desirable hemodynamic unloading of hyperfiltration. If both creatinine and albuminuria rise, afferent restriction may have reduced glomerular pressure without improving downstream peritubular perfusion or tubular protein processing. In such a phenotype, adding RAAS blockade or prioritizing RAASi may be more logical, provided blood pressure and potassium allow it.

## 14. Mechanistic Separation: RAASi, SGLT2i, and ns-MRAs

In the proposed model, RAASi, SGLT2i, and ns-MRAs are not ranked as stronger or weaker agents. Instead, they are positioned according to the pathophysiological node they predominantly address.

RAASi can be conceptualized as efferent/postglomerular therapy. Their key actions include reducing efferent arteriolar tone, intraglomerular pressure, and albuminuria, and possibly improving postglomerular peritubular perfusion. The most logical starting scenarios are A2/A3 albuminuria, elevated blood pressure, pressure-driven phenotype, diabetic kidney disease, glomerular hypertension, and signs of a filtration node.

SGLT2i can be conceptualized as proximal tubular/TGF-afferent therapy. By inhibiting SGLT2, they reduce proximal sodium and glucose reabsorption, increase sodium delivery to the macula densa, restore TGF, increase afferent tone, reduce maladaptive glomerular hyperfiltration, and lower tubular transport work, hypoxia, and volume-metabolic overload. The most logical starting scenarios are diabetes, obesity, heart failure, TGF-mediated hyperfiltration, zero or negative RFR with A1/A2 albuminuria, normal or low-normal blood pressure, and evidence of proximal tubular overload.

Ns-MRA therapy can be conceptualized as anti-inflammatory and anti-fibrotic residual-risk therapy. Finerenone reduces mineralocorticoid-mediated inflammatory and fibrotic processes and has demonstrated clinical benefit in patients with CKD and type 2 diabetes, particularly with persistent albuminuria on background RAAS blockade [24,25,26]. In this algorithm, ns-MRA therapy is most often the third component of staged triple therapy rather than a universal first-line agent.

### 14.1. Rationale for the Placement of Finerenone in the Proposed Framework

Finerenone deserves explicit consideration because FIDELIO-DKD, FIGARO-DKD, and the pooled FIDELITY analysis demonstrated clinically meaningful kidney and cardiovascular benefits in patients with CKD and type 2 diabetes, particularly in the presence of persistent albuminuria and appropriate potassium and eGFR conditions [24,25,26]. Its placement as a later or residual-risk component in this framework should not be interpreted as downgrading the evidence for finerenone. Rather, it reflects the design of the pivotal trials, in which finerenone was generally tested on a background of optimized RAAS blockade and in albuminuric diabetic CKD. Mechanistically, ns-MRA therapy addresses mineralocorticoid-mediated inflammation, fibrosis, vascular dysfunction, and residual cardiorenal risk rather than the immediate afferent or efferent hemodynamic mechanisms emphasized for SGLT2i and RAASi. In a patient with type 2 diabetes, A2/A3 albuminuria, preserved potassium control, and appropriate eGFR, finerenone should be considered according to guideline-supported indications irrespective of whether RFR is measured. What remains hypothesis-generating is whether RFR can help determine the timing and tolerability of adding ns-MRA therapy to RAASi and SGLT2i in complex phenotypes.

### 14.2. Evidence Hierarchy: Established Clinical Evidence Versus Conceptual Extrapolation

The therapeutic sequence proposed in this review should be read through an explicit evidence hierarchy. The strongest clinical evidence supports the use of RAAS blockade in albuminuric and hypertensive CKD, SGLT2 inhibition for reducing CKD progression across broad albuminuria and eGFR strata, and finerenone for patients with type 2 diabetes, CKD, persistent albuminuria, and appropriate potassium and eGFR conditions. These recommendations remain guideline-based and should not be displaced by RFR testing.

By contrast, the choice of RAASi-prioritized versus SGLT2i-prioritized in low-albuminuric CKD with absent reserve, the use of RFR to anticipate eGFR dip, and the use of RFR to guide staged triple renoprotection are conceptual extrapolations from renal physiology, trial subgroup evidence, and real-world clinical reasoning. These elements are proposed as testable hypotheses. They should be used to structure clinical thinking and future research, not as definitive evidence-based directives.

This distinction is clinically important. The model supports guideline-concordant therapy where evidence is established, while using RFR and hemodynamic phenotyping to address the unresolved practical question of therapeutic sequencing in patients who do not fit neatly into trial-derived pathways.

## 15. Three Variants of Low RFR: Filtration, TGF-Proximal, and Mixed Nodes

A central point of the proposed concept is that low RFR indicates exhaustion of functional reserve, but not the mechanism of that exhaustion. The mechanism must be inferred from the accompanying phenotype. Three main patterns can be distinguished.

### 15.1. Filtration/Glomerular Node

This pattern is dominated by injury or overload of the glomerular filtration barrier. Podocytes are injured, the filtration barrier becomes stiff or sclerotic, intraglomerular pressure is high, and afferent-efferent regulation has already reached maximal compensation. Clues include A2/A3 albuminuria, RFR close to zero or negative, normal or reduced eGFR, absent GFR increase after load, and possible hematuria or systemic features of glomerular disease. The therapeutic implication is RAASi priority followed by early SGLT2i addition; in diabetes with persistent albuminuria, ns-MRA therapy may be added when potassium and eGFR allow.

### 15.2. TGF-Proximal/Afferent Node

This is an under-recognized but clinically important pattern. The proximal tubule reabsorbs sodium, glucose, amino acids, and filtered proteins and participates in TGF regulation. When proximal sodium-glucose reabsorption is chronically increased, less sodium reaches the macula densa, TGF signaling is displaced, and the afferent arteriole may remain excessively dilated. The nephron may operate at its limit while RFR is low. Clues include normal or mildly increased UACR, normal or reduced eGFR, RFR close to zero, diabetes, obesity, high sodium intake, volume overload, and urinary tubular markers. This is a TGF-mediated hyperfiltration phenotype, for which SGLT2i-prioritized may be physiologically coherent, followed by RAASi if UACR rises, blood pressure increases, or a pressure phenotype emerges.

### 15.3. Mixed Node

The most severe phenotype combines filtration and TGF-proximal overload. Clues include A2/A3 albuminuria, reduced eGFR, zero or negative RFR, elevated blood pressure, tubular markers, diabetes, obesity, heart failure, tubulointerstitial hypoxia, and high residual risk. The nephron has exhausted reserve in both glomerular and tubular-TGF compartments. The therapeutic implication is not monotherapy but early combined anti-hyperfiltration treatment with RAASi plus SGLT2i, followed by ns-MRA when indicated and safe, with tight monitoring of blood pressure, sodium, weight, and volume status.

## 16. Preserved RFR and the Possibility of Staged Triple Renoprotection

Preserved RFR is not a guarantee of safety, but it can be interpreted as a functional marker of adaptive capacity. In patients with preserved RFR, stable hemodynamics, and indications for combined therapy, staged intensification with RAASi, SGLT2i, and ns-MRA may be undertaken more confidently. This is particularly relevant in diabetes, A2/A3 albuminuria, high cardiorenal risk, and preserved ability to respond to physiological stress.

Every step of intensification should include monitoring of serum creatinine, eGFR, potassium, blood pressure, volume status, UACR, symptoms of hypotension, the eGFR dip at 2–4 weeks, and reassessment at 8–12 weeks. KDIGO recommends monitoring blood pressure, creatinine, and potassium 2–4 weeks after initiating or increasing RAASi; ACE inhibitors or ARBs are generally continued if creatinine rises by no more than 30% within 4 weeks. Ns-MRA therapy requires particularly careful potassium monitoring [1,11,18].

The practical meaning of preserved RFR is not that all agents can be prescribed aggressively at the same time, but that the kidney demonstrates adaptive capacity. Staged intensification is therefore more acceptable when RFR is preserved, whereas in zero or negative RFR, slow titration with one new agent at a time may be safer.

## 17. Proposed Hypothesis-Generating Algorithm for Renal Functional Reserve–Informed Personalized Renoprotection in CKD

A simplified workflow for applying the proposed framework is provided in Box 1.

Box 1 **Simplified clinical flowchart for the proposed framework.**

1. Classify CKD by KDIGO CGA and apply established guideline indications.
2. Confirm persistent albuminuria/proteinuria and exclude physiological, orthostatic, postglomerular, or purely tubular patterns when suspected.
3. Assess blood pressure, volume status, sodium exposure, eGFR slope, diabetes, heart failure, solitary kidney/reduced nephron mass, and medication background.
4. Measure RFR only when the result may clarify whether the kidney has recruitable reserve or is functioning at its adaptive limit.
5. Interpret RFR together with phenotype, not as a standalone threshold.
6. Select or sequence RAASi, SGLT2i, and ns-MRA within guideline-supported indications, using slower titration and closer monitoring when RFR is zero/negative or volume sensitivity is high.

## 18. Practical Interpretation of the Algorithm

The algorithm is intended to support clinical reasoning and study design rather than replace guideline-based indications. In an A1 patient with preserved RFR and stable eGFR, observation and correction of lifestyle, sodium intake, body weight, and blood pressure may be sufficient unless another evidence-based indication for SGLT2i exists. In an A1 patient with zero or negative RFR, normal blood pressure, and a metabolic or reduced-nephron-mass phenotype, SGLT2i-prioritized sequencing should be regarded as a TGF-directed anti-hyperfiltration hypothesis. It should not be used as a stand-alone reason to prescribe SGLT2i in the absence of guideline-supported indications or a research protocol.

In an A2 patient with low RFR, the combination of albuminuria and exhausted reserve may indicate a higher risk and may support consideration of early dual therapy when guideline-based indications, blood pressure, potassium, and volume status permit. In A3 albuminuria, the filtration/pressure component is strong enough that RAAS blockade remains foundational unless contraindicated. SGLT2i may be added early when tolerated, and ns-MRA therapy should be considered in type 2 diabetes with persistent albuminuria, normal potassium, and appropriate eGFR.

The algorithm also provides a framework for interpreting early changes after therapy initiation, including albuminuria changes that may function as surrogate markers of long-term kidney risk [76]. A modest eGFR dip with UACR reduction is often desirable and may indicate hemodynamic unloading. A creatinine rise with stable or reduced urea and reduced UACR may not represent true structural deterioration. A creatinine rise accompanied by increased albuminuria, symptomatic hypotension, volume depletion, or elevated urea requires reassessment of the phenotype, dose, volume status, and drug sequence [77,78,79,80,81].

## 19. Illustrative Therapeutic Trajectories: How the Algorithm May Work in Practice

The following scenarios are illustrative phenotype-based therapeutic vignettes. They combine anonymized real-world clinical experience with didactic hypothetical interpretation and are not intended to represent identifiable case reports, a prospective case series, or evidence of efficacy or harm. Specific values, when mentioned, are approximate didactic examples rather than analyzable patient-level data. Their purpose is solely educational: to show how RFR, UACR, tubular markers, blood pressure, volume status, and early treatment response might be integrated in clinical reasoning. These vignettes should not be used as treatment recommendations without guideline-based indications and prospective validation.

### 19.1. IgA Nephropathy: Creatinine and Albuminuria Increase After SGLT2i

In a patient with IgA nephropathy, empagliflozin initiation may be followed by increased creatinine and increased urinary albumin. If RFR is low or negative, this response may indicate a mixed phenotype in which afferent/TGF modulation reduces glomerular inflow but does not correct peritubular hypoperfusion or tubular protein handling. If blood pressure and potassium allow, adding RAAS blockade or switching priority toward RAASi may be more coherent than simply escalating SGLT2i. If the rise is excessive or accompanied by clinical hypovolemia, temporary discontinuation and re-phenotyping may be necessary.

### 19.2. ADPKD: Creatinine Increase After SGLT2i

In autosomal dominant polycystic kidney disease, architectural distortion and reduced effective nephron mass may produce a low or borderline RFR. SGLT2i initiation may cause a creatinine increase, especially when renal perfusion reserve is limited. This does not necessarily indicate irreversible injury, but it requires careful assessment of volume status, blood pressure, urea, urinary markers, and UACR response. Low-dose initiation may be reasonable in selected patients, but the evidence base for ADPKD remains less robust than for classical diabetic or albuminuric CKD.

### 19.3. Type 1 Diabetes with High RFR: Creatinine Dip Without True Functional Deterioration

In type 1 diabetes with high RFR, SGLT2i may produce a creatinine increase while urea decreases and albuminuria falls. This pattern may represent a beneficial hemodynamic dip rather than true loss of kidney function, particularly if the patient is clinically euvolemic and UACR improves. A high RFR suggests adaptive capacity, making staged combination therapy potentially safer, although SGLT2i use in type 1 diabetes requires careful ketoacidosis risk assessment and adherence to local regulatory recommendations.

### 19.4. Chronic Glomerulonephritis: Different Responses to Dapagliflozin and Empagliflozin

A patient with chronic glomerulonephritis may experience eGFR decline on dapagliflozin but eGFR improvement or stabilization after switching to empagliflozin. This scenario does not prove class heterogeneity in an individual patient, but it illustrates that drug-specific pharmacodynamics, volume effects, adherence, dose, and baseline phenotype may influence response. Repeating RFR before and after switching may help determine whether the second response reflects improved hemodynamic compatibility rather than random variability. Recent observational data comparing dapagliflozin and empagliflozin in advanced CKD support the need for cautious within-class evaluation [82].

### 19.5. Hereditary Nephrotic Syndrome: Borderline RFR and Different Short-Term Responses to SGLT2i

In hereditary nephrotic syndrome with borderline but present RFR, different SGLT2 inhibitors may be associated with different short-term eGFR and UACR responses in clinical practice. This pattern should not be interpreted as proof of within-class superiority. It illustrates that the same mechanistic class may have different tolerability in a given patient depending on dose, volume status, adherence, sodium intake, baseline phenotype, and measurement variability. The therapeutic decision should be based on the integrated response: eGFR slope, UACR reduction, symptoms, blood pressure, potassium, urea, and tubular markers.

### 19.6. Low-Dose SGLT2i in ADPKD

In ADPKD with low or borderline RFR, low-dose SGLT2i may cause only a small creatinine increase. This may be interpreted as a cautious afferent/TGF intervention in a kidney with limited reserve. The aim is not aggressive eGFR reduction but gentle modulation of maladaptive hemodynamics, provided volume status, blood pressure, and symptoms remain stable. This remains a conceptual interpretation because the evidence base for SGLT2i in ADPKD is not comparable to that in diabetic, heart failure, or broad albuminuric CKD populations.

### 19.7. Nephrotic Syndrome: UACR Falls on SGLT2i but Rises After Finerenone

In nephrotic syndrome, SGLT2i may reduce UACR, while subsequent addition of finerenone may be followed by unexpected UACR variability in selected real-world observations. This should not be interpreted as evidence that finerenone increases albuminuria. In randomized trials, finerenone reduced albuminuria and improved cardiorenal outcomes in CKD associated with type 2 diabetes [24,25,26]. The vignette is included only to emphasize that in nephrotic or low-reserve phenotypes, background disease activity, volume status, sodium intake, RAASi dose, potassium constraints, and regression-to-the-mean may complicate interpretation. Such observations support staged therapy and careful monitoring rather than simultaneous multi-drug initiation.

### 19.8. Transplanted Solitary Kidney and Native Solitary Kidney: High Sensitivity to Hemodynamic Intervention

In a transplanted solitary kidney, low-dose empagliflozin may increase creatinine when RFR is low, reflecting the limited adaptive capacity of a single functioning renal unit. Similarly, in a native solitary kidney, finerenone may reduce eGFR and increase urea when the reserve is extremely limited. These examples illustrate why solitary kidney status should be considered a high-sensitivity phenotype. Therapy may still be beneficial, but dosing, sequencing, and monitoring must be conservative.

## 20. A Practical Stepwise Algorithm Based on RFR

A practical workflow may be summarized as follows. First, classify CKD according to KDIGO CGA and confirm persistence of albuminuria or proteinuria. Second, phenotype proteinuria using first-morning urine, UACR, total protein-to-creatinine ratio, albumin-to-total-protein ratio, sediment, and low-molecular-weight proteins. Third, assess eGFR slope, blood pressure, volume status, metabolic phenotype, and nephron-mass context. Fourth, measure RFR under a standardized protocol when the clinical question is whether the kidney is working with reserve or at its limit.

If RFR is preserved, staged intensification may be considered when there are guideline-based indications and hemodynamics are stable. If RFR is zero or negative, therapy should be individualized by phenotype: RAASi-prioritized for pressure/albuminuric patterns, SGLT2i-prioritized for TGF-mediated metabolic or volume phenotypes with low-normal blood pressure, early dual therapy for mixed patterns, and cautious single-agent initiation when hypovolemia or hypotension risk is high.

The early response should be read physiologically. A desired eGFR dip is modest, early, and accompanied by lower albuminuria or improved clinical status. An adverse dip is excessive, progressive, accompanied by rising urea, worsening albuminuria, hypotension, symptoms, or signs of volume depletion. In the latter case, therapy sequence, dose, volume status, and the presumed phenotype should be reconsidered.

## 21. Validation Roadmap, Mechanistic Parallels, and Future Directions

The proposed model requires prospective validation before it can be used as a clinical decision tool. A first validation step would be an observational cohort study enrolling patients across predefined CKD phenotypes: A1 low-albuminuric CKD with diabetes or obesity, A2–A3 albuminuric CKD, solitary kidney or reduced nephron mass, ADPKD, transplant recipients, and patients with uncertain eGFR slope. Each patient would undergo standardized CGA classification, blood pressure assessment, proteinuria phenotyping, tubular marker profiling, and RFR testing before the initiation or intensification of renoprotective therapy. The algorithm in Table 4 and Box 1 should therefore be treated as a study framework: each decision point requires testing against predefined outcomes rather than immediate adoption as a clinical rule.

Primary endpoints should include annualized eGFR slope, change in UACR, occurrence and magnitude of early eGFR dip, tolerability of RAASi/SGLT2i/ns-MRA combinations, hyperkalemia, hypotension, acute kidney injury, and progression to clinically meaningful kidney outcomes. Secondary endpoints may include changes in FLCs, beta2-MG, urinary cystatin C, albumin-to-total-protein ratio, ambulatory blood pressure profiles, sodium sensitivity, and markers of tubular stress or inflammation. Multivariable models should test whether RFR adds independent prognostic information beyond age, sex, baseline eGFR, UACR, blood pressure, diabetes, obesity, and medication exposure.

A second validation step would be an interventional study in which patients are stratified by RFR status and proteinuria phenotype before treatment sequencing. For example, patients with A1–A2 albuminuria and zero or negative RFR could be randomized to SGLT2i-prioritized versus RAASi-prioritized strategies, whereas patients with A2–A3 albuminuria and low RFR could be studied under early dual therapy versus conventional stepwise intensification. Such trials should evaluate not only long-term eGFR slope but also the physiological meaning of early eGFR dip and albuminuria response.

The framework also aligns with broader models of chronic disease progression in which organ injury is sustained by self-reinforcing inflammatory, metabolic, hemodynamic, and barrier-dysfunction loops. Although described in other chronic conditions, including neurodegenerative disease, such systems-level thinking may be useful in CKD, where glomerular barrier stress, proximal tubular overload, peritubular hypoxia, RAAS activation, sodium retention, inflammation, and fibrosis may reinforce one another [83]. In this context, RFR can be viewed as a functional readout of the kidney’s remaining capacity to buffer these loops.

Emerging CKD therapies, including endothelin receptor antagonists, aldosterone synthase inhibitors, GLP-1 receptor agonists, anti-inflammatory strategies, anti-fibrotic approaches, and microbiome-directed interventions, may be incorporated into the same validation logic. The key translational question is not only whether a drug reduces risk in a population, but which pathophysiological node it modifies and whether the individual kidney has sufficient functional reserve to tolerate and benefit from that intervention.

## 22. Limitations of the Concept

This review proposes a conceptual framework rather than a validated clinical prediction model or treatment algorithm. RFR methodology is not yet standardized across centers, and thresholds such as RFR below 5%, zero RFR, or negative RFR require validation. Protein or amino acid loading protocols, timing, dietary state, hydration, background therapy, sodium balance, and GFR measurement method may all influence results [4,5,65,66].

The proposed proteinuria and biomarker phenotyping approach also requires broader implementation of urinary markers that are not routinely measured in many clinics. UACR, total protein, albumin-to-total-protein ratio, beta2-MG, FLCs, cystatin C, and other low-molecular-weight proteins may not be universally available. Assay availability, cost, urine handling, pH sensitivity, reference intervals, and inter-laboratory standardization may limit implementation. Broader biomarkers such as NGAL, KIM-1, TNF receptors, MCP-1, EGF, calprotectin, uromodulin, extracellular vesicle markers, and metabolomic signatures are promising but were not included as core decision nodes because their treatment-predictive role remains insufficiently standardized for routine sequencing of renoprotective therapy [52,53,54].

The two-compartment interpretation of albuminuria is also a conceptual simplification. Rare tubular disorders and experimental models are informative but may not be directly generalizable to common CKD phenotypes. The magnitude of physiological albumin filtration and the relative contribution of tubular reabsorption remain areas of ongoing debate; therefore, the albuminuria section should be read as a mechanistic framework rather than a settled quantitative model.

The clinical vignettes are educational illustrations based on anonymized clinical experience and hypothetical interpretation. They do not constitute a formal case series, do not provide comparative efficacy data, and should not be used to infer benefit or harm of any specific sequence, including finerenone-containing sequences.

Finally, the model does not replace guideline indications. KDIGO-based treatment recommendations remain the foundation. The proposed extension is intended to support personalized sequencing, interpretation of early hemodynamic responses, and hypothesis generation for future clinical studies.

A further limitation is that the proposed model assumes that RFR can be measured reproducibly enough to influence clinical reasoning. This assumption remains to be proven. Until standardized protocols and prospective outcome data are available, RFR should be considered a research-enriched clinical tool and a framework for phenotyping rather than a mandatory parameter for routine CKD management.

Finally, the therapeutic sequencing recommendations in this review intentionally combine established evidence with mechanistic extrapolation. The evidence-supported components are clearly anchored in existing trials and guidelines, whereas RFR-informed sequencing remains a hypothesis requiring prospective validation.

## 23. Conclusions

KDIGO CGA remains the essential starting point for CKD classification, risk assessment, and guideline-based therapy. The proposed renal functional reserve-informed extension should be interpreted as an additional physiological layer, not as a substitute for established recommendations. eGFR and albuminuria alone do not always reveal whether the remaining nephrons are functioning with adaptive reserve or at their physiological limit.

RFR may provide additional physiological information about nephron recruitability and reserve exhaustion, while blood pressure, volume status, and proteinuria phenotype may help contextualize treatment tolerance and early hemodynamic responses. Albuminuria should be interpreted as a two-compartment phenomenon involving both glomerular passage and proximal tubular handling of filtered proteins.

In summary, KDIGO provides the risk map, RFR may show the functional reserve of the nephron, and blood pressure helps determine the hemodynamic safety of treatment intensification. The proposed functional–hemodynamic extension of KDIGO CGA remains a hypothesis-generating framework. It should be used to formulate testable research questions and to support cautious clinical reasoning, not to replace established guideline indications. Before routine clinical implementation, it requires operational standardization, reproducibility testing, and prospective validation in clinically relevant CKD phenotypes.

## Figures and Tables

**Table 1:** Practical phenotyping of proteinuria before renoprotective therapy selection.

Phenotype	Urinary Clues	Clinical Clues	Relationship to RFR and Therapy
Glomerular	Albumin/total protein usually >60–70%; UACR A2–A3; low-molecular-weight proteins absent or only moderately increased	Hematuria, casts, and low serum albumin may be present in nephrotic syndrome	Low RFR with A2–A3 suggests a filtration/pressure phenotype: RAASi-prioritized plus early SGLT2i
Tubular	Albumin/total protein <30–40%; prominent low-molecular-weight proteins such as alpha1-microglobulin, beta2-microglobulin, RBP, cystatin C, or FLC	Serum albumin is often normal; edema is absent; Fanconi syndrome, Dent disease, or drug-related tubular injury may be present	Low RFR plus low-molecular-weight proteinuria suggests tubular overload or a tubulointerstitial node; cautious staged therapy is required
Mixed	High UACR plus increased low-molecular-weight proteins; intermediate albumin/total protein ratio	Glomerular disease with secondary tubular overload or tubulointerstitial injury	High progression risk: early dual therapy; staged triple therapy when indicated and when RFR is preserved or borderline
Postglomerular bleeding	Albumin/total protein approximately 50–60%; low-molecular-weight proteins do not dominate	Macroscopic hematuria, clots, urological symptoms; no dysmorphic erythrocytes or casts	Should not be interpreted as a hyperfiltration phenotype; urological/postglomerular evaluation is required
Orthostatic/physiological	First-morning urine negative; daytime samples positive; total protein may be high during the day	Normal serum albumin, blood pressure, urinary sediment, and eGFR; common in adolescents and young adults	Persistent proteinuria must be confirmed before renoprotection; transient and postural causes should be excluded

**Table 2:** Conceptual interpretation of nephron overload markers.

Marker	Glomerular Component	Tubular Component	Combined Phenotype
ACR	Markedly increased	Mildly increased or unchanged	Markedly increased
FLC	Markedly increased in advanced filtration impairment	Increased with proximal tubular overload	Markedly increased
Beta2-MG	Markedly increased in advanced filtration impairment	Increased with tubular injury or impaired reabsorption	Markedly increased
RFR	0% or negative	0–5%	0% or negative

**Table 3:** Practical checklist for RFR testing and reporting.

Protocol Element	Suggested Reporting Standard	Rationale/Caveat
Baseline state	Clinically stable; no acute illness; document CKD cause, eGFR, UACR, BP, volume status, sodium/protein intake, and recent medication changes.	RFR is highly sensitive to sodium balance, volume status, RAAS tone, glycemia, and background therapy.
Stressor	Prespecify oral protein/meat load, amino acid infusion, or another validated stressor; record dose and composition.	Different stressors produce different peak responses; results should not be compared across protocols without caution.
GFR method	Prefer measured GFR when feasible; if creatinine/cystatin C estimates are used, report this explicitly.	Small short-term changes may be obscured by assay variability or non-GFR determinants.
Timing	Obtain basal value and repeated post-load values over a predefined interval, commonly 2–4 h depending on protocol.	The time to peak response varies between individuals and protocols.
Calculation	Report absolute and relative changes: RFR = stimulated GFR − basal GFR RFR% = [(stimulated GFR − basal GFR) / basal GFR] × 100	Both units are needed because baseline GFR modifies the clinical meaning of the response.
Interpretation	Preserved, low, zero, negative, or <5% RFR should be interpreted within clinical context and not as standalone treatment rules.	Cut-offs in this review are hypothesis-generating and require prospective validation.

**Table 4:** Proposed hypothesis-generating algorithm for selecting initial and subsequent renoprotective therapy according to UACR, RFR, blood pressure, and clinical phenotype. The proposed therapeutic sequences require prospective validation and should be applied only within guideline-based indications and clinical judgment.

UACR, mg/mmol	RFR	BP/Clinical Phenotype	Interpretation	Initial Strategy	Next Step
<3 (A1)	Preserved	Normal BP, stable eGFR, no diabetes, HF, obesity, solitary kidney, or reduced nephron mass	Low current risk; functional reserve preserved	Observation; salt, weight, protein and BP control; repeat UACR/eGFR	SGLT2i only for specific indications: T2D, HF, or adverse eGFR slope
<3 (A1)	Zero or negative	Normal or low-normal BP; diabetes, obesity, solitary kidney, familial risk, or reduced nephron mass	Hidden/relative hyperfiltration: eGFR maintained by maximal nephron workload	Do not prescribe SGLT2i solely because of A1 albuminuria and low RFR; consider SGLT2i-prioritized sequencing only if there is an evidence-based indication (e.g., T2D, HF, reduced eGFR, adverse slope) or within a research protocol.	Add RAASi if UACR rises, BP increases, or pressure phenotype appears; otherwise monitor phenotype and outcomes.
<3 (A1)	Zero or negative	Elevated BP	Hidden hyperfiltration plus systemic/intraglomerular pressure	RAASi-prioritized if BP and potassium allow	Consider SGLT2i only when guideline-supported or clinically justified; monitor creatinine, potassium, BP, and volume status.
<3 (A1)	Preserved	Elevated BP	Pressure-driven phenotype with preserved reserve	RAASi-prioritized	SGLT2i for T2D, HF, reduced eGFR, or adverse slope; RFR supports safer titration
3–30 (A2)	Preserved	Normal or moderately elevated BP	Early albuminuric CKD; reserve still present	RAASi if BP/diabetes; SGLT2i for eGFR/T2D/HF indications	Assess UACR response at 8–12 weeks; combine if reduction is insufficient
3–30 (A2)	Zero or negative	Any BP, especially diabetes, obesity, reduced eGFR, or adverse slope	Albuminuria plus exhausted reserve = high progression risk	Consider early dual therapy when guideline indications, BP, volume status, and potassium permit; order depends on BP, volume, and potassium.	In T2D with persistent UACR >3 mg/mmol, consider ns-MRA
>30 (A3)	Any	Often elevated BP; hematuria or systemic features may be present	Severe albuminuric/glomerular phenotype; barrier injury plus pressure likely	RAASi as foundational therapy + early SGLT2i addition	Exclude GN/podocytopathy; consider biopsy; in T2D consider ns-MRA
>30 (A3)	Zero or negative	Any BP	A3 plus exhausted reserve = maximal risk; nephrons operate at their limit	Consider a combined anti-hyperfiltration strategy when guideline-supported; avoid simultaneous aggressive initiation in unstable patients.	RAASi + SGLT2i, then ns-MRA when indicated and potassium is normal
Any UACR	Preserved	High risk but stable hemodynamics	Functional capacity for intensification exists	Titrate renoprotection more confidently only if guideline indications and stable hemodynamics are present.	Staged triple therapy when indicated: RAASi + SGLT2i + ns-MRA
Any UACR	Zero or negative	Low BP, advanced age, diuretics, HF, or hypovolemia risk	Exhausted reserve plus high risk of excessive eGFR dip	Start with one agent, low dose, slow titration	Monitor at 1–2 and 4 weeks; avoid aggressive simultaneous loading

## Data Availability

No new data were created or analyzed in this study. Data sharing is not applicable to this article.

## Abbreviations

ABPM	ambulatory blood pressure monitoring
ACEi	angiotensin-converting enzyme inhibitor
ACR	albumin-to-creatinine ratio
ADPKD	autosomal dominant polycystic kidney disease
AKI	acute kidney injury
ARB	angiotensin receptor blocker
BP	blood pressure
CGA	cause, glomerular filtration rate, and albuminuria
CKD	chronic kidney disease
CKD-EPI	Chronic Kidney Disease Epidemiology Collaboration
CV	cardiovascular
DKD	diabetic kidney disease
eGFR	estimated glomerular filtration rate
ESKD	end-stage kidney disease
FAS	full age spectrum
FLC	free light chains
RFR	renal functional reserve
GBM	glomerular basement membrane
GFR	glomerular filtration rate
HF	heart failure
IgA	immunoglobulin A
KDIGO	Kidney Disease: Improving Global Outcomes
LMW	low molecular weight
mGFR	measured glomerular filtration rate
MRA	mineralocorticoid receptor antagonist
ns-MRA	non-steroidal mineralocorticoid receptor antagonist
RAAS	renin–angiotensin–aldosterone system
RAASi	renin–angiotensin–aldosterone system inhibitor
RCT	randomized controlled trial
SBP	systolic blood pressure
SGLT2	sodium–glucose cotransporter 2
SGLT2i	sodium–glucose cotransporter 2 inhibitor
T1D	type 1 diabetes
T2D	type 2 diabetes
TGF	tubuloglomerular feedback
UACR	urinary albumin-to-creatinine ratio
UPCR	urinary protein-to-creatinine ratio
β2-MG	beta-2 microglobulin
